# Conditional screening for ultrahigh-dimensional survival data in case-cohort studies

**DOI:** 10.1007/s10985-021-09531-7

**Published:** 2021-08-20

**Authors:** Jing Zhang, Haibo Zhou, Yanyan Liu, Jianwen Cai

**Affiliations:** 1 School of Statistics and Mathematics, Zhongnan University of Economics and Law, Wuhan, 430073, China; 2 Department of Biostatistics, University of North Carolina at Chapel Hill, Chapel Hill, NC 27599-7420, USA; 3 School of Mathematics and Statistics, Wuhan University, Wuhan, 430072, China

**Keywords:** Case-cohort design, Conditional screening, Sure screening property, Survival data, Ultrahigh-dimensional data, Weighted estimating equation

## Abstract

The case-cohort design has been widely used to reduce the cost of covariate measurements in large cohort studies. In many such studies, the number of covariates is very large, and the goal of the research is to identify active covariates which have great influence on response. Since the introduction of sure independence screening (SIS), screening procedures have achieved great success in terms of effectively reducing the dimensionality and identifying active covariates. However, commonly used screening methods are based on marginal correlation or its variants, they may fail to identify hidden active variables which are jointly important but are weakly correlated with the response. Moreover, these screening methods are mainly proposed for data under the simple random sampling and can not be directly applied to case-cohort data. In this paper, we consider the ultrahigh-dimensional survival data under the case-cohort design, and propose a conditional screening method by incorporating some important prior known information of active variables. This method can effectively detect hidden active variables. Furthermore, it possesses the sure screening property under some mild regularity conditions and does not require any complicated numerical optimization. We evaluate the finite sample performance of the proposed method via extensive simulation studies and further illustrate the new approach through a real data set from patients with breast cancer.

## 1 Introduction

In large epidemiological cohort studies, it is common that some diseases of interest (e.g., cancer, heart disease, HIV infection) have very low incidence. In addition, some exposures can be very expensive to measure and it is not feasible to obtain the measures on all cohort members due to restrictions on resources. To reduce the cost while keeping as much efficiency as possible, Prentice (1986) proposed the case-cohort design, where the expensive covariates are obtained only for a random sample of the full cohort, called the subcohort, as well as the additional cases who have experienced the event of interest during the follow-up period. When covariate dimension *p* is smaller than sample size *n*, various methods have been proposed for analyzing data under this design, such as the pseudo-likelihood approach (Prentice, 1986; Self and Prentice, 1988; Kalbfleisch and Lawless, 1988), the estimating equation method (Chen and Lo, 1999; Chen, 2001), the multiple imputation approach (Marti and Chavance, 2011; Keogh and White, 2013), the maximum likelihood estimation (Scheike and Martinussen, 2004; Zeng and Lin, 2014), weighted estimating equation approach (Barlow, 1994; Borgan et al., 2000; Kulich and Lin, 2004; Breslow and Wellner, 2007; Kang and Cai, 2009; Kim et al., 2013), among others.

With the rapid development of biomedical technology, high-dimensional data are frequently collected in large epidemiological studies. The feature of this kind of data is that the covariate dimension *p* is much larger than sample size *n*. An important purpose of analyzing this type of data is to identify a subset of covariates related to the event of interest and construct the effective models based on the selected covariates. For scenarios where *p* increases with *n* at polynomial rate (e.g., *p* = *n^α^* with *α* > 0), the regularization method has been demonstrated to be an effective dimension reduction method for simple random sampling (SRS) data (e.g., Tibshirani, 1996; Fan and Li, 2001; Zou, 2006; Candes and Tao, 2007; Zhang, 2010) and has been generalized to high-dimensional data under the case-cohort design recently. For example, Ni et al. (2016) proposed a variable selection procedure by using the smoothly clipped absolute deviation (SCAD) penalty (Fan and Li, 2001) for scenarios where *p* increases at a slower rate than *n*. Kim and Ahn (2019) proposed a bi-level variable selection method to select non-zero group and within-group variables for cases where variables have group structure. These methods can select variables and estimate parameters simultaneously, however, the computation inherent in regularization methods makes them involve the simultaneous challenges of computational expediency, statistical accuracy, and algorithmic stability when the dimension *p* is ultrahigh in the sense that *p* = exp(*n^α^*) with *α* > 0 (Fan et al., 2009).

For SRS data, the feature screening method has achieved great success in dealing with the challenge of ultrahigh-dimensional settings. Various marginal screening methods have been proposed under different settings, such as linear models (Fan and Lv, 2008), generalized linear models (Fan and Song, 2010), additive models (Fan et al., 2011), the varying coefficient models (Fan et al., 2014; Liu et al., 2014) and model-free scenarios (e.g., Zhu et al., 2011; Li et al., 2012a; Li et al., 2012b; He et al., 2013; Chang et al., 2013; Cui et al., 2015; Mai and Zou, 2015; Wu and Yin, 2015). For censored survival data, several model-based screening methods (e.g., Tibshirani, 2009; Zhao and Li, 2012; Gorst-Rasmussen and Scheike, 2013) and model-free screening methods (e.g., Song et al., 2014; Wu and Yin, 2015; Zhang et al., 2017; Zhou and Zhu, 2017; Liu et al., 2018; Zhang et al., 2018; Lin et al., 2018; Pan et al., 2019) have been proposed via defining different marginal utilities. Although they are powerful in reducing the dimensionality, they may face some challenges in some situations. For instance, as noted in Fan and Lv (2008), the correlation among covariates heavily influence the marginal utility. When the correlation among covariates is relatively high, the marginal screening methods may fail to retain the hidden active variables which have great influence on response but are weakly correlated with the response. Although some iterative screening methods (e.g., Fan and Lv, 2008; Zhu et al., 2011; Zhang et al., 2018, Pan et al., 2019) and forward screening approaches (e.g., Wang, 2009) have been proposed to alleviate this problem, the computation speed is relatively slow and the statistical properties are elusive.

In many applications, researchers can obtain some prior information of active variables from previous investigations and experiences. For example, in the breast cancer study (van de Vijver et al., 2002), gene AL080059 has been known to be predictive to patients’ survival time in the literature (Yeung et al., 2005; van’t Veer et al., 2002). Barut et al. (2016) pointed out we can improve the accuracy in variable screening by including such prior knowledge. In view of this thought, they proposed the conditional screening approach for generalized linear models and showed that conditioning helps reducing the correlation among covariates, thus can detect the hidden active variables with higher probability. Hong et al. (2016) further proposed to integrate prior information using data-driven approaches. Hu and Lin (2017) put forward a conditional screening procedure via ranking covariates based on conditional marginal empirical likelihood ratios. Liu and Wang (2017) proposed a screening method based on conditional distance correlation. Hong et al. (2018) developed a conditional screening method for censored data under the proportional hazards model. Liu and Chen (2018) considered the conditional quantile independence screening approach for ultrahigh-dimensional heterogeneous data. Lu and Lin (2020) proposed a model-free conditional screening via conditional distance correlation. Extensive simulation studies showed these conditional screening methods which incorporate important prior information of active variables can provide a powerful means to identify hidden active variables for ultrahigh-dimensional data.

The research on marginal and conditional screening methods has been fruitful for ultrahigh-dimensional SRS data, but to the best of our knowledge, conditional screening method has not been studied for case-cohort data, the existing conditional screening methods can not be directly applied to the case-cohort data due to its special data structure. To fill the gap, we propose a conditional screening method for ultrahigh-dimensional case-cohort data under the framework of Cox proportional hazards model. We construct the marginal hazards regression models for each covariate by including the known important covariates. As some covariates are not fully observed, we build the weighted estimating equation to obtain the estimators of the parameters. Then we propose the marginal utilities based on the parameter estimates to measure the contribution of each covariate and retain the covariates with top ranked contributions. We refer to it as conditional weighted screening method, in short the C-WSIS procedure. As pointed out by Barut et al. (2016), the correlation between covariates can be weakened upon conditioning, so that hidden active covariates have a higher chance to be retained. Therefore, the proposed method enables the detection of hidden active covariates for ultrahigh dimensional survival data under the case-cohort design. Under some reasonable conditions, it enjoys the sure screening property and the ranking consistency. Our research is the first one that focus on conditional screening for ultrahigh dimensional case-cohort data, it can be viewed as an extension of Hong et al. (2018) from SRS data to case-cohort data. Note that although the ideas are similar, the generalization is quite challenging due to the much more complex structure of case-cohort data, both implementation and the theory will be quite different.

The rest of the article is organized as follows. In Section 2, we introduce the model, data and present the details of the CWSIS procedure. In Section 3, we establish the theoretical properties of the proposed CWSIS method. Section 4 presents results from simulation studies. A real data set from the breast cancer study is analyzed in Section 5. Section 6 provides some remarks and discussions. The regularity conditions and the technical proofs are presented in the Appendix.

## 2 Conditional screening for case-cohort data

Suppose there are *n* independent subjects in a cohort study. Let *T_i_* and *C_i_* denote the failure time and censoring time of subject *i*, we only observe *X_i_* = min(*T_i_*, *C_i_*) and *Δ_i_* = *I*(*T_i_* ≤ *C_i_*) due to right-censoring. Let **Z**_*i*_ = (*Z*_*i*1_,…,*Z_ip_*)^T^ denote the *p*-dimensional covariate, under the case-cohort design, **Z**_*i*_ is available only on the cases (*Δ_i_* = 1) and the subcohort (a random subset of the full cohort). Let *ξ_i_* be the indicator for subcohort membership, i.e., *ξ_i_* = 1 and 0 denote whether or not the *i*th subject in the full cohort is selected into the subcohort. For the selection of subcohort, we consider independent Bernoulli sampling with selection probability *π* = *Pr*(*ξ_i_* = 1) ∈ (0, 1). Thus, the observable data for the *i*th subject is {*X_i_*, *Δ_i_*, **Z**_*i*_, *ξ_i_*} when *ξ_i_* = 1 or *Δ_i_* = 1, and {*X_i_*, *Δ_i_*, *ξ_i_*} when *ξ_i_* = 0 and *Δ_i_* = 0.

Suppose that the failure time follows the proportional hazards model (Cox, 1972), under which the conditional hazard function of *T_i_* given **Z**_*i*_ has the form

(1) $$\lambda \left(t|Z_{i}\right)=\lambda_{0}(t)\exp\left(\alpha^{\text{T}}Z_{i}\right),$$

where λ_0_(*t*) is the unspecified baseline hazard function and ***α*** = (*α*_1_,…,*α_p_*)^T^ is the unknown regression parameter. Assume that the failure time *T_i_* and the censoring time *C_i_* are independent given **Z**_*i*_. In an ultrahigh-dimensional setting, the dimensionality *p* greatly exceeds sample size *n* and can be allowed to increase at an exponential rate of *n*. Under the sparsity principle, only a small number of covariates have great influence on the response variable, i.e., ‖***α***‖ is much smaller than *p*, where ‖***α***‖ denotes the number of nonzero elements of ***α***. Assume we have the prior information that a set of covariates are related to survival time *T* and the index set is denoted by C,q=|C| denotes the number of covariates in *C*. Write $Z_{i,C}=\left(Z_{i,j},j\in C\right),Z_{i,-C}=\left(Z_{i,j},j\notin C\right),\alpha_{C}=\left(\alpha_{j},j\in C\right)$ and $\alpha_{-C}=\left(\alpha_{j},j\notin C\right)$. Here, C is known, $\alpha_{C}$ and $\alpha_{-C}$ are unknown. The true hazard function in (1) is equivalent to

(2) $$\lambda \left(t|Z_{i}\right)=\lambda_{0}(t)\;\exp\left(\alpha_{C}^{\text{T}}Z_{i,C}+\alpha_{-C}^{\text{T}}Z_{i,-C}\right).$$

Let $A_{-C}=\left\{j\notin C\;:\;\alpha_{j}\neq 0\right\}$ and $a=\left|A_{-C}\right|=\sum_{j\notin C}\;I\left(\alpha_{j}\neq 0\right)$ be the true set of non-zero coefficients and its cardinality. Our goal is to recover the set $A_{-C}$ as precisely as possible based on data from case-cohort studies. In other words, we want to find a subset of covariates ${\hat{A}}_{-C}$ which satisfies $A_{-C}\subseteq {\hat{A}}_{-C}$.

To perform an initial screening procedure, we construct the marginal Cox regression models for each covariate individually, here we also add the known covariates in C to each marginal model. Specifically, for j∉C the hazard function of *T_i_* given ($Z_{i,C}$, *Z_i,j_*) has the form

(3) $$\lambda \left(t|Z_{i,C},Z_{i,j}\right)=\lambda_{j,0}(t)\;\exp\left(\beta_{C,j}^{\text{T}}Z_{i,C}+\beta_{j}Z_{i,j}\right),$$

where λ_*j*,0_(*t*) is the unspecified baseline hazard function, and $\beta_{C,j}$ and *β_j_* are the unknown regression parameters corresponding to covariates $Z_{C}$ and *Z_j_* in the marginal Cox model, respectively. Since the covariates can only be observed for the selected subcohort and cases for case-cohort data, we consider the following weighted estimating equation

(4) $$U_{j}\left(\beta_{C,j},\beta_{j}\right)={\left[U_{j,k}\left(\beta_{C,j},\beta_{j}\right),k\in C\cup \{j\}\right]}^{\text{T}}=0_{q+1},$$

with

$$U_{j,k}\left(\beta_{C,j},\beta_{j}\right)=\overset{n}{\underset{i=1}{\sum }}\int_{0}^{\tau }\left\{Z_{ik}-\frac{{\tilde{S}}_{j,k}^{\left(1\right)}\left(\beta_{C,j},\beta_{j},t\right)}{{\tilde{S}}_{j,k}^{\left(0\right)}\left(\beta_{C,j},\beta_{j},t\right)}\right\}\;\text{d}N_{i}(t)=0,$$

where ${\tilde{S}}_{j,k}^{\left(l\right)}\left(\beta_{C,j},\beta_{j},t\right)=n^{-1}\sum_{i=1}^{n}Z_{i,k}^{l}w_{i}(t)Y_{i}(t)\;\exp\left(\beta_{C,j}^{\text{T}}Z_{i,C}+\beta_{j}Z_{i,j}\right)$ for k∈C∪{j} and *l* = 0, 1, 2. Here, we choose the time-varying weight function $w_{i}(t)=\Delta_{i}+\left(1-\Delta_{i}\right)\xi_{i}/\hat{\pi }(t)$, where $\hat{\pi }(t)=\sum_{i=1}^{n}(1-\Delta_{i})\xi_{i}Y_{i}(t)/\sum_{i=1}^{n}\left(1-\Delta_{i}\right)Y_{i}(t)$ is a consistent estimator of the true sampling probability *π*. Note that *w_i_*(η) weights the *i*th subject by the inverse probability of selection, it equals to 1 for the cases and $\hat{\pi }{\left(t\right)}^{-1}$ for the sampled censored subjects. The maximum marginal pseudo-partial likelihood estimator $\left({\hat{\beta }}_{C,j},{\hat{\beta }}_{j}\right)$ is defined as the solution to the weighted estimating equation $U_{j}\left(\beta_{C,j},\beta_{j}\right)=0_{q+1}$. Define the information matrix $I_{j}\left(\beta_{C,j},\beta_{j}\right)=-{\left(\partial U_{j,k}\left(\beta_{C,j},\beta_{j}\right)/\partial \beta_{l}\right)}_{k,l\in C\cup \{j\}}$ which is of (*q*+1) dimension. Let ${\hat{\sigma }}_{j}^{2}={\left[I_{j}\left({\hat{\beta }}_{C,j},{\hat{\beta }}_{j}\right)\right]}_{q+1,q+1}^{-1}$ be the variance estimate of ${\hat{\beta }}_{j}$, i.e., the (*p* + 1)th diagonal element of matrix $I_{j}\left({\hat{\beta }}_{C,j},{\hat{\beta }}_{j}\right)$. For j∉C, we define

$$M_{C,j}=\frac{\left|{\hat{\beta }}_{j}\right|}{{\hat{\sigma }}_{j}},$$

which serves as the proposed utility measure for the *j*th covariate. We rank covariates *Z_j_* (j∉C) by the value of $M_{C,j}$ from the largest to smallest and retain those at the top of the rank list. For a given threshold *γ* > 0, the selected index set in addition to set C is given by

(5) $${\hat{A}}_{-C}=\left\{j\notin C\;:\;M_{C,j}=\frac{\left|{\hat{\beta }}_{j}\right|}{{\hat{\sigma }}_{j}}\geq \gamma \right\}.$$

In practical applications, we can pre-determine a positive integer *d*_0_ and define the estimated active set as

$${\hat{A}}_{-C}=\left\{j\;:\;M_{C,j}\;\text{is amongst the first}\;d_{0}\;\text{largest of all}\;M_{C,j}\;(j\notin C)\right\}.$$

Similar to Fan and Lv (2008) and other literature related to feature screening, we can choose *d*_0_ = ⌈*n_cc_*/log *n_cc_*⌉, where *n_cc_* denotes the case-cohort sample size.

**Fig. 1:**
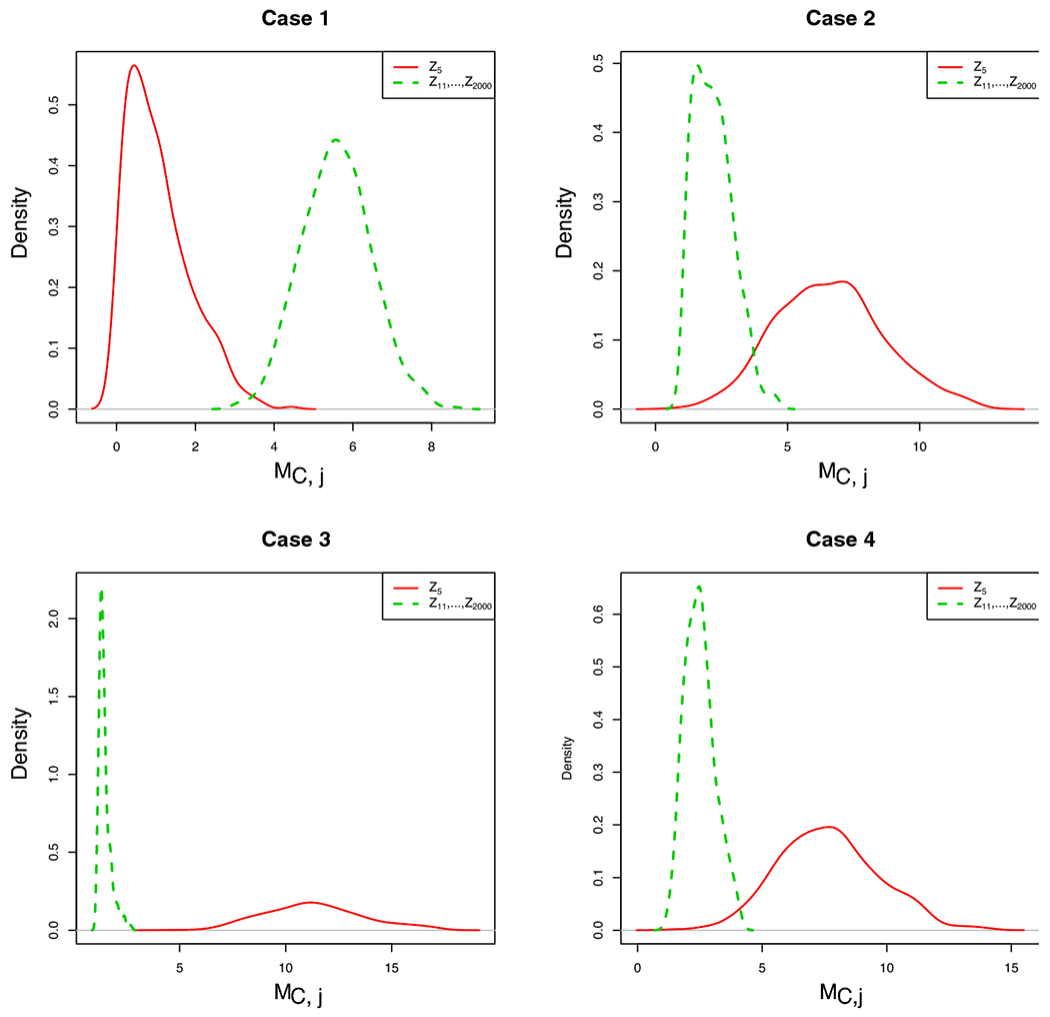
Density of the screening statistic $M_{C,j}$ for the hidden active covariate *Z*_5_ compared with a mixture of densities of inactive covariates *Z*_6_, …, *Z*_2000_ with different conditioning sets: Case 1: C = {∅} which is equivalent to marginal screening; Case 2: C = {1}, one truly active covariate; Case 3: C = {1, 2}, two truly active covariates; Case 4: C = {6, 7, 8}, three inactive covariates. The full cohort sample size *n* = 500, number of covariates *p* = 2000, noncase-to-case ratio is 1 : 1, the failure rate equals to 20%.

Similar to the conditional screening procedures of Barut et al. (2016) and Hong et al. (2018), the outstanding advantage of the proposed CWSIS procedure is that it enables the detection of hidden active covariates for ultrahigh dimensional case-cohort data. To demonstrate this merit, we set up an example in a similar way to Barut et al. (2016) and Hong et al. (2018). In particular, the failure time *T_i_* follows the Cox proportional hazards model $\lambda \left(t|Z_{i}\right)=\lambda_{0}(t)\;\exp\left(\alpha^{\text{T}}Z_{i}\right)$, where λ_0_(*t*) = 1, $\alpha ={\left(1_{4}^{\text{T}},-2,0_{p-5}^{\text{T}}\right)}^{\text{T}}$, **Z**_*i*_ ~ *N_p_*(**0**, *Σ*) with *Σ* = (*σ_ij_*)_*p*×*p*_, *σ_ii_* = 1 for *i* = 1,…,*p*, *σ_ij_* = 0.5 for *i* ≠ *j*. By this design, *Z*_5_ is a hidden active covariate. We consider four different conditioning sets, C = {∅}, {1}, {1, 2}, {6, 7, 8}. The densities of the proposed screening statistic $M_{C,j}$ for *Z*_5_ (hidden active covariate) and *Z*_6_, …, *Z*_2000_ (inactive covariates) are summarized in Figure 1. When C=∅, CWSIS is equivalent to the marginal screening approach, the value of $M_{C,j}$ for *Z*_5_ is much smaller than the corresponding value of inactive covariates with a high probability. When the conditioning set includes one truly active covariate (C={1}), the curve for *Z*_5_ is on the right and there is a clear separation between these two curves. When we include more truly active covariates (C={1,2}), this separation becomes larger. We note a very interesting phenomenon that when the conditioning set consists of three inactive covariates (C={6,7,8}), the chance of identifying the hidden variable *Z*_5_ using CWSIS is still higher than the marginal screening method. This may be due to the correlation between them and the active covariates, such inactive variables can effectively function as surrogates for the active variables, thus conditioning on them can help detect hidden variables. A similar phenomenon was also observed in Barut et al. (2016) and Hong et al. (2018).

## 3 Theoretical property

In this section, we show the CWSIS procedure enjoys the sure screening property and the ranking consistency property, which demonstrate that our CWSIS procedure tends to rank the active covariates above the inactive ones with high probability, furthermore, all the active covariates survive after screening with probability tending to 1 as *n* → ∞. These two properties lay out the theoretical foundation of our CWSIS procedure. Define $S_{k}^{\left(l\right)}(t)=n^{-1}\sum_{i=1}^{n}Z_{ik}^{l}Y_{i}(t)\lambda \left(t|Z_{i}\right),s_{k}^{\left(l\right)}(t)=E\left\{S_{k}^{\left(l\right)}(t)\right\},S_{j,k}^{\left(l\right)}\left(\beta_{C,j},\beta_{j},t\right)=n^{-1}\sum_{i=1}^{n}Z_{i,k}^{l}Y_{i}(t)\;\exp\left(\beta_{C,j}^{\text{T}}Z_{i,C}+\beta_{j}Z_{i,j}\right)$ and $s_{j,k}^{\left(l\right)}\left(\beta_{C,j},\beta_{j},t\right)=E\left\{S_{j,k}^{\left(l\right)}\left(\beta_{C,j},\beta_{j},t\right)\right\}$ for k∈C∪{j} and *l* = 0, 1, 2. Let ${\left(\beta_{C,j}^{0},\beta_{j}^{0}\right)}^{\text{T}}$ be the solution of the following equation $u_{j}\left(\beta_{C,j},\beta_{j}\right)={\left[u_{j,k}\left(\beta_{C,j},\beta_{j}\right),k\in C\cup \{j\}\right]}^{\text{T}}=0_{q+1}$, with

$$u_{j,k}\left(\beta_{C,j},\beta_{j}\right)=\int_{0}^{\tau }\left\{s_{k}^{\left(1\right)}(t)-\frac{s_{j,k}^{\left(1\right)}\left(\beta_{C,j},\beta_{j},t\right)}{s_{j,k}^{\left(0\right)}\left(\beta_{C,j},\beta_{j},t\right)}s_{k}^{\left(0\right)}(t)\right\}\;\text{d}t=0.$$

The regularity conditions are given in Appendix A, under which we establish the following lemmas and theorems.

**Lemma 1** *Under conditions C1-C8*, $\beta_{j}^{0}=0$ *if and only if α_j_* = 0 *for all* j∉C.

**Lemma 2** *Suppose conditions C1-C8 hold, there exist constants c*_2_ > 0 *and* 0 < *κ* < 1/2 *such that*

$$\underset{j\in A_{-C}}{\min}\left|\beta_{j}^{0}\right|\geq c_{2}n^{-\kappa }.$$

**Lemma 3** *Under conditions C1-C8, for any ϵ*_1_ > 0 *and ϵ*_2_ > 0, *there exist positive constant c*_3_ *and integer N such that for any n* > *N and* 0 < *κ* < 1/2,

$$P\left(\underset{j\in A_{-C}}{\max}\left|{\hat{\beta }}_{j}-\beta_{j}^{0}\right|>c_{2}\left(n^{-\kappa }+\epsilon_{1}\right)/2\right)\leq 2a(q+1)\;\exp\left(-c_{3}n^{1-2\kappa }\right)+a\epsilon_{2},$$

*where a is the size of* $A_{-C}$, *q is the size of* C, *c*_2_ *is the same value in lemma 2*.

Lemma 3 shows that the proposed maximum marginal pseudo-partial likelihood estimate ${\hat{\beta }}_{j}$ is a consistent estimate of $\beta_{j}^{0}$. By lemmas 1 and 3, we indeed can distinguish $Z_{j}\left(j\in A_{-C}\right)$ from $Z_{j}\left(j\notin A_{-C}\right)$ by the proposed marginal utility $M_{C,j}$. Theorem 1 states the sure independent screening property of the CWSIS procedure.

**Theorem 1 (The sure screening property)** *Under conditions C1-C8, for any* 0 < *κ* < 1/2 *and ϵ*_2_ > 0, *there exists positive constant c*_3_ *such that*

$$P\left(A_{-C}\subseteq {\hat{A}}_{-C}\right)\geq 1-2a(q+1)\;\exp\left(-c_{3}n^{1-2\kappa }\right)-a\epsilon_{2},$$

where a is the size of $A_{-C}$, q is the size of C. Furthermore, we have

$$\underset{n\to \infty }{\lim}P\left(A_{-C}\subseteq {\hat{A}}_{-C}\right)=1.$$

From this theorem, we can see that all active covariates survive after screening with a probability tending to one. The next theorem establishes the ranking consistency property of the proposed method.

**Theorem 2 (The ranking consistency)** *Under conditions C1-C8, we have*

$$P\left(\underset{j\notin A_{-C}}{\max}M_{C,j}<\underset{j\in A_{-C}}{\min}M_{C,j}\right)\to 1$$

*when n* → ∞.

This lays out the theoretical foundation that our procedure ensures active covariates be ranked prior to the inactive ones with overwhelming probability. The proof of theorems and these lemmas are presented in the Appendix B.

## 4 Simulation studies

We examine the finite sample performance of the proposed CWSIS procedure and make comparisons with some existing methods via simulation studies. For brevity, we refer to the feature aberration at survival times screening procedure of Gorst-Rasmussen and Scheike (2013) as FAST-SIS, the principled sure independent screening procedure of Zhao and Li (2012) as P-SIS, the censored rank independence screening of Song et al. (2014) as CRIS. Furthermore, we consider the marginal weighted screening procedure (MWSIS), where we fit the marginal Cox regressions $\lambda \left(t|Z_{ij}\right)=\lambda_{0j}^{*}(t)\;\exp\left(\beta_{j}Z_{ij}\right)$ for each *Z_ij_* and construct the weighted estimating equation to obtain the estimate ${\hat{\beta }}_{j}$, then define the active index set as $\hat{A}=\left\{1\leq j\leq p\;:\;\left|{\hat{\beta }}_{j}\right|I_{j}{\left({\hat{\beta }}_{j}\right)}^{1/2}\geq \gamma \right\}$, *I_j_*(*β_j_*) denotes the information matrix. As the PSIS, FAST and CRIS can only deal with the SRS data, we generate the SRS data with the same sample size as the case-cohort data for PSIS, FAST and CRIS.

We consider the survival data generated from the Cox proportional hazards model and employ the independent Bernoulli sampling to generate the subcohort. We consider full cohort sample size *n* = 500, 1000, and the number of covariates *p* = 2000, 4000. As the incidence rate for case-cohort studies is usually very low or moderate, we consider the failure rate of 20% for *n* = 500, 5% and 10% for *n* = 1000. We consider the noncase-to-case ratio of 1 : 1, thus the sample size of the case-cohort data in our simulation studies equals to 100, 200. For each configuration, we repeat 500 simulations and employ three evaluation criteria (Li et al., 2012b). The first one is the minimum model size to include all active predictors, denoted by S. We present the median and interquartile range (IQR) of S out of 500 replications. The second one is the selection proportion that each important variable is selected into the model with a given model size *d*_0_, denoted by $P_{e}$. The third one is the selection proportion that all important variables are selected into the model with a given model size *d*_0_, denoted by $P_{a}$. An effective screening procedure is expected to yield S close to the true minimum model size and both $P_{e}$ and $P_{a}$ close to one. Here, we choose *d*_0_ = ⌈*n_cc_*/log *n_cc_*⌉ (Fan and Lv, 2008), *n_cc_* is the case-cohort sample size and ⌈*x*⌉ denotes the integer part of *x*.

### Example 1.

*T_i_* are generated from the Cox proportional hazards model

$$\lambda \left(t|Z_{i}\right)=\lambda_{0}(t)\;\exp\left(\alpha^{\text{T}}Z_{i}\right),$$

where $\lambda_{0}(t)=1,\alpha ={\left(1_{4}^{\text{T}},-2,0_{p-5}^{\text{T}}\right)}^{\text{T}},Z_{i}∼N_{p}(0,\Sigma )$ with $\Sigma ={\left(\sigma_{ij}\right)}_{p\times p},\sigma_{ii}=1$ for *i* = 1,…*p, σ_ij_* = 0.5 for *i* ≠ *j*. The censoring time *C_i_* ~ Unif(0, *τ*), the constant *τ* represents the end time of the study and is used to control the failure rate.

### Example 2.

We consider the same model as example 1, with $\alpha ={\left(10,0_{p-2}^{\text{T}},1\right)}^{\text{T}}$, i.e., only *Z*_1_ and *Z_p_* are active covariates. The first (*p* − 1) covariates $\left(Z_{1},\ldots ,Z_{\left(p-1\right)}\right)∼N_{p-1}(0,\Sigma )$ with $\Sigma ={\left(\sigma_{ij}\right)}_{\left(p-1)\times (p-1\right)}$, where *σ_ii_* = 1 for *i* = 1, …, (*p* − 1), *σ_ij_* = *ρ* for *i* ≠ *j*. We vary the value of *ρ* to be 0, 0.3, 0.7, with a larger *ρ* yielding a higher collinearity. The last covariate *Z_p_* ~ *N*(0, 1).

**Fig. 2:**
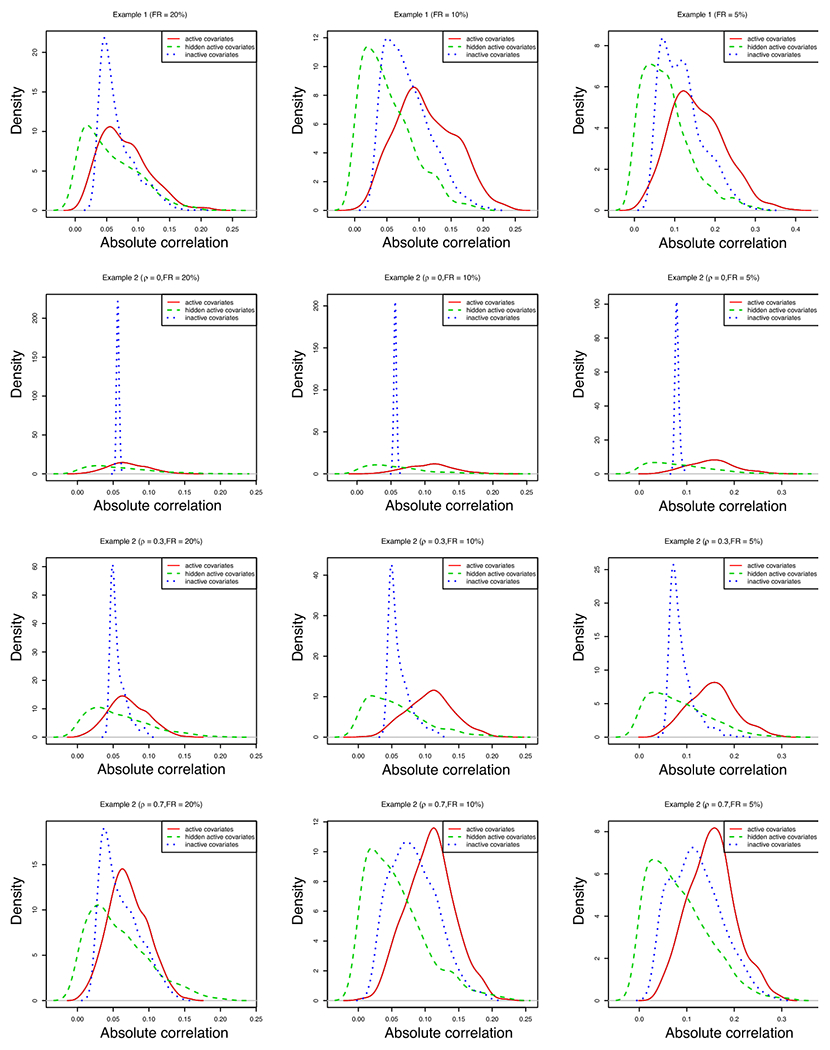
Absolute correlation of the survival time and the covariates for *p* = 2000.

**Fig. 3:**
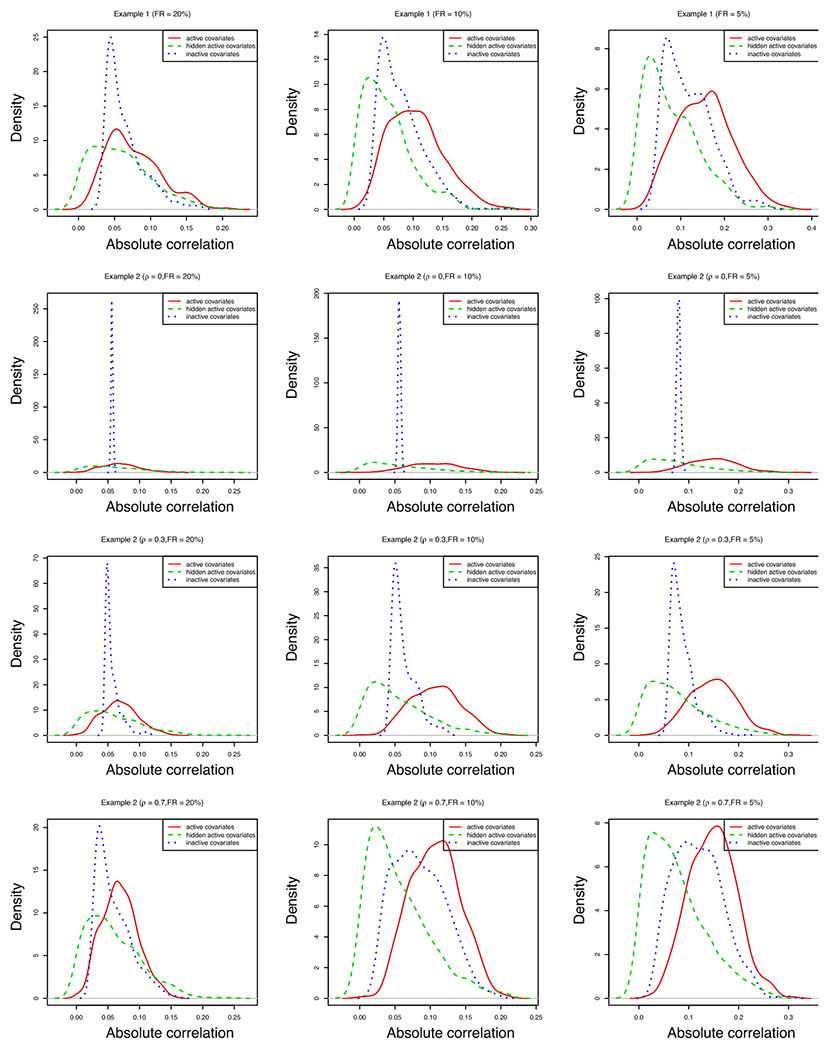
Absolute correlation of the survival time and the covariates for *p* = 4000.

We compute the absolute correlation between the survival time *T* and each covariate *Z_j_* (*j* = 1, …, *p*) for *p* = 2000 through the inverse probability weighting scheme and further summarize the marginal correlation in three groups: the active covariates (*Z*_1_, …, *Z*_4_ for example 1 and *Z*_1_ for example 2), the hidden active covariates (*Z*_5_ for example 1 and *Z_p_* for example 2), and the inactive covariates (*Z*_6_, …, *Z_p_* for example 1 and *Z*_2_, …, *Z*_(*p*−1)_ for example 2). Figures 2 and 3 depict the distribution of the absolute correlation for these three groups, from which we can see the marginal signal strength of hidden active covariates are weaker than the inactive covariates. Therefore, the marginal screening methods MWSIS, PSIS, FAST and CRIS are difficult to identify the hidden active covariates. The proposed conditional screening method CWSIS is an ideal alternative. In our simulations, we simply choose *Z*_1_ as the conditional covariate. In practice, if we have no useful prior information about active covariates, we can choose those covariates which have higher marginal signal strength as the conditional set (Barut et al., 2016; Lu and Lin, 2020). To have a fair comparison, we add one (the number of conditional covariate in our examples) to S for the proposed conditional screening method CWSIS.

The simulation results for S, $P_{e}$ and $P_{a}$ are summarized in Tables 1–2. By observing the values of $P_{e}$ for *Z*_5_ in example 1 and *Z_p_* in example 2, we can conclude that the proposed CWSIS procedure can detect the hidden active covariates with high probabilities, while the other four methods MWSIS, PSIS, FAST and CRIS fail to select them. In example 2, *ρ* equals to 0, 0.3, and 0.7, with a larger *ρ* yielding a higher collinearity. The proposed method CWSIS performs well even with high collinearity, while the other four methods do not behave well even when *ρ* = 0 and the performance deteriorates with the increasing value of *ρ*. As expected, CWSIS needs a smaller model size to possess the sure screening property in all settings. Larger case-cohort sample size and higher failure rate are associated with better performance. In particular, larger cohort sample size can handle rare disease situations better.

To assess the performance of the proposed method in the settings that are similar to the real data, we further consider *n* = 300 and the failure rate of 25% for example 2, the remaining setups are kept the same as before. Here, we also consider the unweighted conditional screening method NCWSIS which does not adopt the weight function and simply treat the case-cohort data as SRS data, and the conditional screening method C-SMPLE in Hong et al. (2018). Since the method C-SMPLE in Hong et al. (2018) is proposed for SRS data, it can not be directly used to handle the case-cohort data, we generate the SRS data with the same sample size as the case-cohort data for CSMPLE. The simulation results for S, $P_{e}$ and $P_{a}$ are summarized in Table 3, from which we can see that the proposed method can detect the hidden active covariates with high probabilities and delivers its distinctive advantages for all the considered settings. By comparing the results of NCWSIS, CSMPLE and CWSIS, we can conclude that the performance of the conditional screening method is improved by including the case-cohort weight. Moreover, the proposed conditional screening procedure based on case-cohort design is more accurate in selecting the active covariates than the conditional screening based on a SRS of the same size as the case-cohort sample. For example, when *p* = 2000 and *ρ* = 0.7, the value of $P_{a}$ is only 0.460 for CSMPLE, while the corresponding value of the proposed method CWSIS equals to 1.

## 5 Application to breast cancer data

As an illustration, we apply the proposed CWSIS method to the breast cancer data (van de Vijver et al., 2002), with 295 female patients who have primary invasive breast carcinoma. For each patient, the expressions of 24885 genes were profiled on cDNA arrays from all tumors. A set of 4919 candidate genes were selected after initial screening using the Rosetta error model (van’t Veer et al., 2002). By excluding the individuals with missing values, we have 289 subjects with 4919 candidate genes. The median observed time was 7.23 years (ranging from 0.05 to 18.34 years). During the follow-up, 78 patients died of breast cancer and the other 211 patients were still alive, which led to the failure rate of 26.99%. Of the 289 patient samples, 60 samples overlapped with the 78 training samples from van’t Veer et al (2002), we use these 60 samples as the testing set and the case-cohort samples as our training set. The details of these two sets are summarized in Table 4. The interest of the study is to identify genes that have great influence on patients’ overall survival rate.

We illustrate the proposed method by identifying genes that have great influence on patients’ overall survival rate based on data from a case-cohort sample. Specifically, we select the subcohort by independent Bernoulli sampling with the selection probability *π* = 0.37, which results in about the same number of cases and noncases. The subcohort has 111 subjects and the final case-cohort sample has 155 subjects. Gene AL080059 has been known to be predictive to patients’ survival time in the literature (Yeung et al., 2005; van’t Veer et al., 2002), we use it as the conditional variable in the proposed procedure. The screening methods are usually considered as an initial step to reduce the dimensionality and then followed with some model-based regularization methods. In particular, we first apply the proposed CWSIS procedure to reduce the dimension from *p* = 4919 to ⌈155/log(155)⌉ = 31 and then utilize different regularization methods LASSO, SCAD and MCP to select the significant ones among these 31 genes under the framework of the Cox proportional hazards regression, the tuning parameter was selected by the 10-fold cross-validation. We summarize the name and the corresponding estimated value of the coefficient for selected genes in Table 5, from which we can see that genes Contig58368.RC, NM.014889, NM.005689, NM.013290, AL080059, NM.013332, Contig63649.RC and NM.002916 were all selected by the LASSO, SCAD and MCP methods, indicating that these eight genes could be associated with patients’ survival rate. Moreover, genes Contig58368.RC, NM.014889 and NM.005689 were ranked at the first three position, which means that these three genes may have great influence on patients’ survival rate.

To evaluate the predictive accuracy of C-WSIS, we further compute the *C*-statistic estimator (Uno et al., 2011). For comparison, we also apply the MWSIS and NC-WSIS procedures to analyze this data. In particular, we first apply these three screening methods to reduce the dimension to ⌈155/log(155)⌉ = 31, then perform the LASSO penalization to further remove some irrelevant covariates, with the tuning parameter selected by the 10-fold cross-validation. We obtain the risk score for each subject by using the final model selected by LASSO and further compute the corresponding concordance statistic (*C*-statistic) (Uno et al., 2011) in the testing set. The standard deviations (SD) of *C*-statistic are obtained from perturbation resampling 1000 times. The corresponding values of *C*-statistic and SD (the values in the parenthesis) are 0.862 (0.059), 0.796 (0.078), 0.802 (0.053) for CWSIS, MWSIS, NCWSIS procedures, respectively. According to Uno et al. (2011), the larger the *C*-statistic is, the stronger predictive power the method possesses. We can conclude that the proposed CWSIS method performs reasonably well for ultrahigh-dimensional survival data under the case-cohort design and delivers a favorable performance in terms of prediction.

We also consider *d_n_* = *n*/2, *n*/3, *n*/4 when analyzing this data and summarize the results in the supplementary material, from which we can see that the selected genes under different cut-offs are highly consistent. Furthermore, we compute the *C*-statistic estimator for CWSIS, MWSIS, NCWSIS procedures under these three cases. From the results in the supplementary material we can make similar conclusion to that with *d_n_* = *n*/log(*n*).

## 6 Conclusion

For ultrahigh-dimensional survival data under the case-cohort design, we propose a conditional screening procedure CWSIS by incorporating the prior information of active covariates. This method enables the detection of hidden active covariates, which is an outstanding advantage compared with the marginal screening procedures. Moreover, the proposed procedure does not require any complicated numerical optimization and is computationally efficient. Theoretically, it enjoys the sure screening property and ranking consistency property under some mild regularity conditions. In the development of the theoretical properties, we adopt the conditional linear expectation and conditional linear covariance, which are proposed in Hong et al. (2018) and are useful to specify the regularity conditions.

There are some issues that deserve further considerations. First, the proposed method requires the prior information of active covariates, sometimes it may be difficult to obtain such useful information. Hong et al. (2016) proposed a data-driven method to obtain the conditional set for generalized linear models. How to develop a data-driven conditional screening method for survival data under the case-cohort is an interesting question. Furthermore, when we have prior knowledge of active covariates, how to balance it with the information extracted from the given data merits further investigation. Second, under our design, the subcohort is selected by independent Bernoulli sampling. When the subcohort is selected by simple random sampling without replacement, our method also works, although more complicated arguments would be needed to develop the theoretical properties. Moreover, when some covariates are available for all cohort members, we can consider the stratified case-cohort design based on those covariates. Third, we can consider to propose more efficient screening methods which incorporating more complex prior knowledge, such as the network structure or the spatial information of the covariates.

## Supplementary Material

1745068_Sup_material

## Figures and Tables

**Table 1:** The median and interquartile range (IQR) of S, the selection proportions $P_{e}$ and $P_{a}$ among 500 replications for example 1

							$P_{e}$					
*p*	*n*	FR	*n_c_*	Method	Median	IQR	*X*_1_	*X*_2_	*X*_3_	*X*_4_	*X*_5_	$P_{a}$
2000	1000	5%	50	PSIS	1849	469	0.130	0.106	0.094	0.102	0.016	0.000
				FAST	1843	446	0.116	0.108	0.106	0.104	0.016	0.000
				CRIS	1398	764	0.290	0.270	0.288	0.250	0.220	0.084
				MWSIS	2000	2	0.354	0.342	0.352	0.342	0.000	0.000
				CWSIS	447	746	–	0.318	0.350	0.330	0.476	0.018
	1000	10%	100	PSIS	1998	18	0.488	0.456	0.436	0.462	0.000	0.000
				FAST	1998	19	0.474	0.444	0.422	0.450	0.000	0.000
				CRIS	1721	379	0.050	0.032	0.020	0.038	0.004	0.000
				MWSIS	2000	0	0.784	0.804	0.774	0.794	0.000	0.000
				CWSIS	69	172	–	0.790	0.768	0.810	0.760	0.356
	500	20%	100	PSIS	2000	1	0.686	0.706	0.706	0.668	0.002	0.000
				FAST	2000	1	0.654	0.654	0.694	0.622	0.002	0.000
				CRIS	1720	405	0.054	0.054	0.044	0.054	0.002	0.000
				MWSIS	2000	0	0.812	0.832	0.840	0.798	0.000	0.000
				CWSIS	47	168	–	0.828	0.852	0.806	0.764	0.442
4000	1000	5%	50	PSIS	3747	716	0.384	0.364	0.376	0.380	0.002	0.000
				FAST	3748	744	0.368	0.352	0.378	0.362	0.002	0.000
				CRIS	3133	1100	0.022	0.018	0.014	0.022	0.000	0.000
				MWSIS	4000	3	0.670	0.700	0.710	0.702	0.000	0.000
				CWSIS	908	1477	–	0.720	0.680	0.734	0.688	0.252
	1000	10%	100	PSIS	3995	46	0.384	0.364	0.376	0.380	0.002	0.000
				FAST	3995	48	0.368	0.352	0.378	0.362	0.002	0.000
				CRIS	3363	795	0.022	0.018	0.014	0.022	0.000	0.000
				MWSIS	4000	0	0.670	0.700	0.710	0.702	0.000	0.000
				CWSIS	136	389	–	0.720	0.680	0.734	0.688	0.252
	500	20%	100	PSIS	4000	2	0.600	0.608	0.578	0.630	0.000	0.000
				FAST	4000	2	0.582	0.592	0.574	0.582	0.000	0.000
				CRIS	3447	871	0.036	0.050	0.038	0.024	0.000	0.000
				MWSIS	4000	0	0.770	0.732	0.730	0.766	0.000	0.000
				CWSIS	86	277	–	0.770	0.784	0.806	0.746	0.350

*n*, the sample size of the full cohort; *p*, the number of covariates; FR, the failure rate; *n_c_*, the average number of cases; CWSIS: the proposed conditional screening method; MWSIS: the marginal weighted screening procedure; PSIS: the screening procedure of Zhao and Li (2012); FAST: the screening procedure of Gorst-Rasmussen and Scheike (2013); CRIS: the screening procedure of Song et al. (2014).

**Table 2:** The median and interquartile range (IQR) of S, the selection proportions $P_{e}$ and $P_{a}$ among 500 replications for example 2

					*p* = 2000					*p* = 4000				
							$P_{e}$					$P_{e}$		
*n*	FR	*ρ*	*n_c_*	Method	Median	IQR	*X*_1_	*X_p_*	$P_{a}$	Median	IQR	*X*_1_	*X_p_*	$P_{a}$
500	20%	0	100	PSIS	578	979	1.000	0.092	0.092	1279	2088	1.000	0.066	0.066
				FAST	594	975	1.000	0.090	0.090	1286	2054	1.000	0.068	0.068
				CRIS	841	1005	1.000	0.032	0.032	1683	1920	0.998	0.014	0.014
				MWSIS	424	951	1.000	0.104	0.104	936	1819	1.000	0.088	0.088
				CWSIS	2	0	–	1.000	1.000	2	0	–	1.000	1.000
		0.3	100	PSIS	1973	131	1.000	0.000	0.000	3958	302	1.000	0.000	0.000
				FAST	1971	138	1.000	0.000	0.000	3958	311	1.000	0.000	0.000
				CRIS	1278	1192	0.998	0.022	0.022	2795	2220	0.998	0.012	0.012
				MWSIS	1997	33	1.000	0.000	0.000	3993	62	1.000	0.002	0.002
				CWSIS	2	0	–	1.000	1.000	2	0	–	1.000	1.000
		0.7	100	PSIS	2000	0	0.380	0.000	0.000	4000	0	0.278	0.000	0.000
				FAST	2000	0	1.000	0.000	0.000	4000	0	1.000	0.000	0.000
				CRIS	1945	460	0.990	0.008	0.008	3923	848	0.978	0.004	0.004
				MWSIS	2000	0	0.676	0.000	0.000	4000	0	0.558	0.000	0.000
				CWSIS	2	0	–	1.000	1.000	2	0	–	1.000	1.000
1000	10%	0	100	PSIS	664	1033	1.000	0.064	0.064	1385	2072	1.000	0.038	0.038
				FAST	684	1024	1.000	0.064	0.064	1376	2031	1.000	0.036	0.036
				CRIS	937	982	0.920	0.014	0.008	1938	2140	0.816	0.002	0.000
				MWSIS	315	795	1.000	0.170	0.170	599	1766	1.000	0.116	0.116
				CWSIS	2	0	–	0.998	0.998	2	0	–	0.998	0.998
		0.3	100	PSIS	1928	374	0.964	0.002	0.002	3870	710	0.946	0.000	0.000
				FAST	1926	382	1.000	0.002	0.002	3863	678	1.000	0.000	0.000
				CRIS	1233	1119	0.884	0.034	0.030	2427	2355	0.810	0.024	0.012
				MWSIS	1999	15	1.000	0.000	0.000	3998	63	1.000	0.000	0.000
				CWSIS	2	0	–	1.000	1.000	2	0	–	0.998	0.998
		0.7	100	PSIS	2000	0	0.042	0.000	0.000	4000	0	0.016	0.000	0.000
				FAST	2000	0	0.996	0.000	0.000	4000	0	0.994	0.000	0.000
				CRIS	1737	930	0.794	0.028	0.024	3451	1921	0.710	0.024	0.010
				MWSIS	2000	0	0.208	0.000	0.000	4000	0	0.150	0.000	0.000
				CWSIS	2	0	–	1.000	1.000	2	0	–	0.998	0.998
1000	5%	0	50	PSIS	1075	984	0.254	0.006	0.002	2010	2274	0.266	0.008	0.006
				FAST	931	1022	0.990	0.008	0.008	1771	2138	1.000	0.002	0.002
				CRIS	1332	1002	0.562	0.000	0.000	2364	1826	0.436	0.000	0.000
				MWSIS	520	983	1.000	0.042	0.042	1082	2023	1.000	0.030	0.030
				CWSIS	2	1	–	0.936	0.936	2	2	–	0.882	0.882
		0.3	50	PSIS	1678	667	0.080	0.002	0.000	3459	1476	0.046	0.004	0.002
				FAST	1580	825	0.958	0.002	0.002	3249	1642	0.976	0.002	0.002
				CRIS	1501	1077	0.568	0.006	0.004	2592	2036	0.448	0.004	0.000
				MWSIS	1981	155	0.984	0.000	0.000	3971	204	0.956	0.000	0.000
				CWSIS	2	0	–	0.946	0.946	2	1	–	0.890	0.890
		0.7	50	PSIS	2000	2	0.000	0.000	0.000	4000	1	0.000	0.000	0.000
				FAST	2000	2	0.502	0.000	0.000	4000	1	0.536	0.000	0.000
				CRIS	1721	1037	0.590	0.010	0.002	3093	2247	0.462	0.008	0.002
				MWSIS	2000	0	0.020	0.000	0.000	4000	0	0.006	0.000	0.000
				CWSIS	2	0	–	0.942	0.942	2	1	–	0.900	0.900

*n*, the sample size of the full cohort; *p*, the number of covariates; FR, the failure rate; *n_c_* the average number of cases; *ρ*, the correlation coefficient of covariates; CWSIS: the proposed conditional screening method; MWSIS: the marginal weighted screening procedure; PSIS: the screening procedure of Zhao and Li (2012); FAST: the screening procedure of Gorst-Rasmussen and Scheike (2013); CRIS: the screening procedure of Song et al. (2014).

**Table 3:** The median and interquartile range (IQR) of S, the selection proportions $P_{e}$ and $P_{a}$ among 500 replications for example 2 with *n* = 300 and FR=25%

			*p* = 2000					*p* = 4000				
					$P_{e}$					$P_{e}$		
*ρ*	*n_c_*	Method	Median	IQR	*X*_1_	*X_p_*	$P_{a}$	Median	IQR	*X*_1_	*X_p_*	$P_{a}$
0	75	PSIS	671	1040	1.000	0.062	0.062	1226	1920	1.000	0.032	0.032
		FAST	711	1035	1.000	0.068	0.068	1221	1927	1.000	0.034	0.034
		CRIS	854	1064	1.000	0.026	0.026	1626	2057	0.998	0.012	0.012
		MWSIS	599	1057	1.000	0.058	0.058	1014	1900	1.000	0.040	0.040
		NCWSIS	5	53	-	0.706	0.706	8	115	-	0.648	0.648
		CSMPLE	7	68	-	0.674	0.674	12	108	-	0.608	0.608
		CWSIS	2	0	-	1.000	1.000	2	0	-	0.994	0.994
0.3	75	PSIS	1960	236	1.000	0.000	0.000	3917	485	1.000	0.000	0.000
		FAST	1959	236	1.000	0.000	0.000	3915	516	1.000	0.000	0.000
		CRIS	1339	1216	0.998	0.014	0.014	2718	2467	0.994	0.020	0.020
		MWSIS	1987	106	1.000	0.000	0.000	3966	225	1.000	0.000	0.000
		NCWSIS	3	60	-	0.712	0.712	4	57	-	0.704	0.704
		CSMPLE	10	65	-	0.648	0.648	16	223	-	0.574	0.574
		CWSIS	2	0	-	1.000	1.000	2	0	-	1.000	1.000
0.7	75	PSIS	2000	0	0.596	0.000	0.000	4000	0	0.578	0.000	0.000
		FAST	2000	0	1.000	0.000	0.000	4000	0	1.000	0.000	0.000
		CRIS	1948	399	0.990	0.002	0.002	3921	1035	0.978	0.006	0.006
		MWSIS	2000	0	0.610	0.000	0.000	4000	0	0.556	0.000	0.000
		NCWSIS	2	40	-	0.732	0.732	2	38	-	0.736	0.736
		CSMPLE	44	204	-	0.460	0.460	74	606	-	0.388	0.388
		CWSIS	2	0	-	1.000	1.000	2	0	-	1.000	1.000

*n*, the sample size of the full cohort; *p*, the number of covariates; FR, the failure rate; *n_c_*, the average number of cases; *ρ*, the correlation coefficient of covariates; PSIS: the screening procedure of Zhao and Li (2012); FAST: the screening procedure of Gorst-Rasmussen and Scheike (2013); CRIS: the screening procedure of Song et al. (2014); MWSIS: the marginal weighted screening procedure; NCWSIS: the unweighted conditional screening method; CSMPLE: the conditional screening method of Hong et al. (2018); CWSIS: the proposed conditional screening method.

**Table 4:** Summary of the breast cancer data

Dataset	Num	Min	Max	Median	Fail(%)
Train	289	0.055	18.341	7.225	26.99
Test	60	0.712	15.352	7.606	38.33

Train, the training set; Test, the testing set; Num, the number of patients; Min, the minimum observed survival time; Max, the maximum observed survival time; Median, the median of observed survival time; Fail, the failure rate.

**Table 5:** The results of selected important genes for the breast cancer data using the regularization methods

LASSO		SCAD		MCP	
Name	Est.	Name	Est.	Name	Est.
Contig58368.RC	0.392	Contig58368.RC	0.516	Contig58368.RC	0.515
NM.014889	0.277	NM.014889	0.446	NM.014889	0.445
NM.005689	0.201	NM.005689	0.329	NM.005689	0.329
NM.013332	0.199	NM.013290	0.326	NM.013290	0.325
Contig63649.RC	0.178	AL080059	0.312	AL080059	0.312
NM.013290	0.172	NM.013332	0.256	NM.013332	0.256
AL080059	0.168	Contig63649.RC	0.249	Contig63649.RC	0.249
NM.002916	0.140	NM.002916	0.204	NM.002916	0.206
NM.012291	0.102				
Contig31288.RC	0.083				
Contig38288.RC	0.049				
NM.003376	0.017				
NM.001673	0.014				

Name: the name for selected genes; Est.: the corresponding estimated value of the coefficient for selected genes.

## Appendix A: regularity conditions

Let $S_{T}\left(t|Z_{i}\right)=\exp\left\{-\Lambda_{0}(t)\;\exp\left(\alpha^{\text{T}}Z_{i}\right)\right\}$ and $S_{C}\left(t|Z_{i}\right)=P\left(C_{i}>t|Z_{i}\right)$ denote the survival functions of *T_i_* and $C_{i},F_{T}\left(t|Z_{i}\right)=1-S_{T}\left(t|Z_{i}\right),\Lambda_{0}(t)=\int_{0}^{t}\lambda_{0}(s)\text{d}s$ denotes the cumulative baseline hazard function. For any vector $\nu =\left(\nu_{1},\ldots ,\nu_{p}\right)\in R^{p}$, let $\|\nu {\|}_{d}=\sqrt[{d}]{\sum_{j=1}^{p}{\left|\nu_{j}\right|}^{d}}$ be the *L_d_* norm. For any random variables $\zeta \;:\;\Omega \to R^{d},\zeta_{1}\;:\;\Omega \to R^{d_{1}},\zeta_{2}\;:\;\Omega \to R^{d_{2}}$ and *η* : *Ω* → *R^p^*, the conditional linear expectation of *ζ* given *η* is defined as $E^{*}(\zeta |\eta )=E(\zeta )+B^{T}\{\eta -E(\eta )\}$, where $B={\mathrm{argmin}}_{D\in R^{d}\times R^{p}}E\left[{\left\{\zeta -E(\zeta )-D^{\text{T}}(\eta -E(\eta ))\right\}}^{2}|\eta \right]$. The conditional linear covariance between *ζ*_1_ and *ζ*_2_ given *η* is defined as $Cov^{*}\left(\zeta_{1},\zeta_{2}|\eta \right)=E^{*}[\{\zeta_{1}-E^{*}\left(\zeta_{1}|\eta \right)\}\left\{\zeta_{2}-E^{*}\left(\zeta_{2}|\eta \right)\right\}|\eta ]$. The properties of *E**(*ζ*|*η*) and *Cov**(*ζ*_1_, *ζ*_2_|*η*) are presented in Appendix B. The regularity conditions listed below are imposed throughout our discussions.

C1. For each j∉C and k∈C∪{j}, there exists a neighborhood $B_{j}$ of ${\left(\beta_{C,j}^{0},\beta_{j}^{0}\right)}^{\text{T}}$ such that

$$\underset{t\in [0,\tau ],{\left(\beta_{C,j},\beta_{j}\right)}^{\text{T}}\in B_{j}}{\sup}\|S_{j,k}^{\left(l\right)}\left(\beta_{C,j},\beta_{j},t\right)-s_{j,k}^{\left(l\right)}\left(\beta_{C,j},\beta_{j},t\right){\|}_{2}\to 0$$

in probability as $n\to \infty (l=0,1),s_{j,k}^{\left(0\right)}\left(\beta_{C,j},\beta_{j},t\right)$ is bounded away from zero on $B_{j}\times [0,\tau ],s_{j,k}^{\left(l\right)}\left(\beta_{C,j},\beta_{j},t\right)$ are bounded on $B_{j}$ × [0, *τ*].

C2. For all $j=1,\ldots ,p,\int_{0}^{\tau }\lambda_{j,0}(t)\text{dt}<\infty$ and E{Y(τ)}>0.

C3. The covariates *Z_j_* (*j* = 1, …, *p*) are independent of time and bounded by a constant *L*_0_. Furthermore, *E*(*Z_j_*) = 0 for all *j* ∈ {1, …, *p*}.

C4. All *Z_j_*, $j\in A_{-C}$ are independent of all *Z_j_*, $j\notin A_{-C}$ given $Z_{C}$.

C5. There exists a constant *L*_1_ such that ‖***α***‖_1_ < *L*_1_ and $\|{\left(\beta_{C,j},\beta_{j}\right)}^{\text{T}}{\|}_{1}<L_{1}$.

C6. There exist constants *c*_1_ > 0 and 0 < *κ* < 1/2 such that $\min_{j\in A_{-C}}|E[Cov^{*}(Z_{j},P(\delta =1|Z)|Z_{C})]|\geq c_{1}n^{-\kappa }$.

C7. There exists a constant *L* > 0 such that $n^{-1}\|U_{j}({\hat{\beta }}_{C,j},{\hat{\beta }}_{j})-U_{j}(\beta_{C,j}^{0},\beta_{j}^{0}){\|}_{2}\geq L\|{\left({\hat{\beta }}_{C,j},{\hat{\beta }}_{j}\right)}^{\text{T}}-{\left(\beta_{C,j}^{0},\beta_{j}^{0}\right)}^{\text{T}}{\|}_{2}$ for all j∉C.

C8. Let $\tilde{n}=\sum_{i=1}^{n}\xi_{i}$ denote the sample size of subcohort, then *ñ/n* converges to the constant *π* ∈ (0, 1).

Conditions C1 and C2 are common assumptions in survival analysis (Andersen and Gill, 1982; Fleming and Harrington, 1991). Condition C3 assumes the covariates are bounded, similar condition also used in Hong et al. (2018). Condition C4 is similar to the partial orthogonality assumption of the covariates. Condition C5 controls the total effect size of the covariates, it is reasonable under the sparsity principle. Condition C6 is a typical assumption which has been widely used in the literature of feature screening, such as condition 3 in Fan and Lv (2008), condition 2 in Li et al. (2012b), condition 2 in Song et al. (2014), conditions 2 and 5 in Wu and Yin (2015), etc. Condition C7 is a mild assumption which holds in many situations. Condition C8 is a common assumption on the case-cohort design.

## Appendix B: lemmas and theoretic proofs

Let $\beta_{C,0}$ be the solution of the equations $u_{C}\left(\beta_{C}\right)={\left[u_{j,k}\left(\beta_{C},0\right),k\in C\right]}^{\text{T}}=0_{q}$. Define $v_{j}\left(\beta_{C,j},\beta_{j}\right)=u_{j,j}\left(\beta_{C,j},\beta_{j}\right)-\underset{k\in C}{\sum }b_{k}u_{j,k}\left(\beta_{C,j},\beta_{j}\right)$, where vector $b_{C}={\left[b_{k},k\in C\right]}^{\text{T}}$ such that $E^{*}\left[Z_{j}|Z_{C}\right]=\underset{k\in C}{\sum }b_{k}Z_{k}$. As a preparation, we first introduce some lemmas.

**Lemma 4** *Let* ***ξ*** = (*ξ*_1_, …, *ξ_n_*) *be a random, vector containing ñ ones and n* − *ñ zeros, with each permutation equally likely. Let B_i_*(*t*) (*i* = 1, …, *n*) be independent and identically distributed real-valued random processes on [0, *τ*] with *E*{*B_i_*(*t*)} = *μ_B_*(*t*), *var*(*B_i_*(*τ*)) < ∞. *Let B*(*t*) = {*B*_1_(*t*), …, *B_n_*(*t*)} *be independent, of ξ*. *Suppose that almost all paths of B_i_*(*t*) *have finite variation. Then*, $n^{-1/2}\sum_{i=1}^{n}\xi_{i}\left\{B_{i}(t)-\mu_{B}(t)\right\}$ *converges weakly in l*^∞^[0, *τ*] *to a zero-mean Gaussian process and therefore* $n^{-1/2}\sum_{i=1}^{n}\xi_{i}\left\{B_{i}(t)-\mu_{B}(t)\right\}$ *converges in probability to zero uniformly in t*.

This Lemma is the same as Lemma A1 of Kang and Cai (2009).

**Lemma 5** *Given that ξ is independent of Δ and Y*(*t*), $n^{1/2}\left\{{\hat{\pi }}^{-1}(t)-\pi^{-1}\right\}$ *converges weakly to a zero-mean Gaussian process*.

This lemma is extracted from lemma A3 of Ni et al. (2016).

**Lemma 6** *For independent random variables Y*_1_, …, *Y_n_ with bounded ranges* [−*M, M*] *and zero mean*,

$$P\left(\left|Y_{1}+\ldots +Y_{n}\right|>y\right)\leq 2\;\exp\;\left(-\frac{1}{2}\frac{y^{2}}{V+My/3}\right)$$

*for V* ≥ *Var*(*Y*_1_ + … + *Y_n_*).

This lemma is extracted from lemma 2.2.9 of van der Vaart and Wellner (1996).

**Lemma 7** *Let ζ*, *ζ*_1_, *ζ*_2_ *and η be any four random variables in the probability space* (*Ω*, F, *P*), the following properties hold for the conditional linear expectation *E**(⋅|*η*) *given η:*

1. $E^{*}(\zeta |\eta )=E(\zeta )+Cov(\zeta ,\eta )Var{\left(\eta \right)}^{-1}\{\eta -E(\eta )\}$;
2. $E^{*}(\eta |\eta )=\eta$;
3. *For any matrices A*_1_ *and A*_2_, $E^{*}\left(A_{1}\zeta_{1}+A_{2}\zeta_{2}|\eta \right)=A_{1}E^{*}\left(\zeta_{1}|\eta \right)+A_{2}E^{*}\left(\zeta_{2}|\eta \right)$;
4. $E^{*}\left[E^{*}(\zeta |\eta )\right]=E\left[E^{*}(\zeta |\eta )\right]=E[\zeta ]$.

This lemma is extracted from proposition 2 of Hong et al. (2018).

**Lemma 8** *The conditional linear covariance has the following properties:*

1. $Cov^{*}\left(\zeta_{1},\zeta_{2}|\eta \right)=0\Leftrightarrow E^{*}\left(\zeta_{1}\zeta_{2}|\eta \right)=E^{*}\left(\zeta_{1}|\eta \right)E^{*}\left(\zeta_{2}|\eta \right)$;
2. $E\left[Cov^{*}\left(\zeta_{1},\zeta_{2}|\eta \right)\right]=Cov\left(\zeta_{1},\zeta_{2}\right)-Cov\left(\zeta_{1},\eta \right)Var{\left(\eta \right)}^{-1}Cov\left(\eta ,\zeta_{2}\right)$;
3. *For any increasing function h*(·) : *R* → *R and random variable ξ* : *Ω* → *R, we have* $Cov^{*}(h(\xi ),\xi |\eta )\geq 0$.

This lemma is extracted from proposition 3 of Hong et al. (2018).

Proof of Lemma 1

*Proof* We first relate $\beta_{j}^{0}\;\text{to}\;E\left[Cov^{*}\left\{Z_{j},P(\delta =1|Z)|Z_{C}\right\}\right]$, then by condition C6, we relate it to *α_j_*. For any j∉C and k∈C, straightforward calculations entail that $s_{k}^{l}(t)=E\left\{Z_{k}^{l}\lambda_{0}(t)\;\exp\left(\alpha^{\text{T}}Z\right)S_{T}S_{C}\right\}$ and $s_{j,k}^{\left(l\right)}\left(\beta_{C,j},\beta_{j},t\right)=E\left\{Z_{k}^{l}\;\exp\left(Z_{C}^{\text{T}}\beta_{C,j}+Z_{j}\beta_{j}\right)S_{T}S_{C}\right\}\;(l=0,1,2)$, then

$$u_{j,k}\left(\beta_{C,j},\beta_{j}\right)=\int_{0}^{\tau }\;E\left\{\;[Z_{k}-\frac{E\left\{Z_{k}\;\exp\left(Z_{C}^{\text{T}}\beta_{C,j}+Z_{j}\beta_{j}\right)S_{T}S_{C}\right\}}{E\left\{\exp\left(Z_{C}^{\text{T}}\beta_{C,j}+Z_{j}\beta_{j}\right)S_{T}S_{C}\right\}}]\;\lambda_{0}(t)\;\exp\left(\alpha^{\text{T}}Z\right)S_{T}S_{C}\right\}\;\text{d}t.$$

By the definition, we have

$$v_{j}\left(\beta_{C,j},\beta_{j}\right)=u_{j,j}\left(\beta_{C,j},\beta_{j}\right)-\underset{k\in C}{\sum }b_{k}u_{j,k}\left(\beta_{C,j},\beta_{j}\right)\equiv F_{1j}\left(\beta_{C,j},\beta_{j}\right)-F_{2j}\left(\beta_{C,j},\beta_{j}\right),$$

where

$$F_{1j}\left(\beta_{C,j},\beta_{j}\right)=\int_{0}^{\tau }\;E\left\{\left(Z_{j}-\underset{k\in C}{\sum }b_{k}Z_{k}\right)\lambda_{0}(t)\;\exp\left(\alpha^{\text{T}}Z\right)S_{T}S_{C}\right\}\text{d}t\;=\int_{0}^{\tau }\;E\left[\left\{Z_{j}-E^{*}\left(Z_{j}|Z_{C}\right)\right\}\lambda_{0}(t)\;\exp\left(\alpha^{\text{T}}Z\right)S_{T}S_{C}\right]\text{d}t\;=E\left[Cov^{*}\left\{Z_{j},P(\delta =1|Z)|Z_{C}\right\}\right],$$

and

$$F_{2j}\left(\beta_{C,j},\beta_{j}\right)\;=\int_{0}^{\tau }\left[\frac{E\left\{Z_{j}\;\exp\left(Z_{C}^{\text{T}}\beta_{C,j}+Z_{j}\beta_{j}\right)S_{T}S_{C}\right\}}{E\left\{\exp\left(Z_{C}^{\text{T}}\beta_{C,j}+Z_{j}\beta_{j}\right)S_{T}S_{C}\right\}}-\underset{k\in C}{\sum }b_{k}\frac{E\left\{Z_{k}\;\exp\left(Z_{C}^{\text{T}}\beta_{C,j}+Z_{j}\beta_{j}\right)S_{T}S_{C}\right\}}{E\left\{\exp\left(Z_{C}^{\text{T}}\beta_{C,j}+Z_{j}\beta_{j}\right)S_{T}S_{C}\right\}}\right]\;\times E\left\{\lambda_{0}(t)\;\exp\left(\alpha^{\text{T}}Z\right)S_{T}S_{C}\right\}\;\text{d}t\;=\int_{0}^{\tau }\frac{E\left[\left\{Z_{j}-E^{*}\left(Z_{j}|Z_{C}\right)\right\}\;\exp\left(Z_{C}^{\text{T}}\beta_{C,j}+Z_{j}\beta_{j}\right)S_{T}S_{C}\right]}{E\left\{\exp\left(Z_{C}^{\text{T}}\beta_{C,j}+Z_{j}\beta_{j}\right)S_{T}S_{C}\right\}}\times E\left\{\lambda_{0}(t)\;\exp\left(\alpha^{\text{T}}Z\right)S_{T}S_{C}\right\}\;\text{d}t.$$

By the definition of $\left(\beta_{C,j}^{0},\beta_{j}^{0}\right)$, we have $u_{j}\left(\beta_{C,j}^{0},\beta_{j}^{0}\right)=0_{q+1}$, then $u_{j,k}\left(\beta_{C,j}^{0},\beta_{j}^{0}\right)=0$ for any k∈C∪{j}, $v_{j}\left(\beta_{C,j}^{0},\beta_{j}^{0}\right)=u_{j,j}\left(\beta_{C,j}^{0},\beta_{j}^{0}\right)-\underset{k\in C}{\sum }b_{k}u_{j,k}\left(\beta_{C,j}^{0},\beta_{j}^{0}\right)=0,F_{2j}\left(\beta_{C,j}^{0},\beta_{j}^{0}\right)=F_{1j}\left(\beta_{C,j}^{0},\beta_{j}^{0}\right)=E[Cov^{*}\{Z_{j},P(\delta =1|Z)|Z_{C}\}]$. When $\alpha_{j}=0,\;E\left[Cov^{*}\left\{Z_{j},P(\delta =1|Z)|Z_{C}\right\}\right]=0$, thus $F_{2j}\left(\beta_{C,j}^{0},\beta_{j}^{0}\right)=0$. Because of $F_{2j}\left(\beta_{C,0},0\right)=0,v_{j}\left(\beta_{C,0},0\right)=E\left[Cov^{*}\left\{Z_{j},P(\delta =1|Z)|Z_{C}\right\}\right]-F_{2j}\left(\beta_{C,0},0\right)=0_{q+1}$. By the uniqueness of the solution of $v_{j}(\beta_{C},\beta )$, we have $\beta_{j}^{0}=0$.

When *α_j_* ≠ 0, by condition C6, we have $F_{2j}\left(\beta_{C,j}^{0},\beta_{j}^{0}\right)=E\left[Cov^{*}\left\{Z_{j},P(\delta =1|Z)|Z_{C}\right\}\right]\geq c_{1}n^{-\kappa }$. This implies that $F_{2j}\left(\beta_{C,j}^{0},\beta_{j}^{0}\right)$ and $E\left[Cov^{*}\left\{Z_{j},P(\delta =1|Z)|Z_{C}\right\}\right]$ are both nonzero and have the same signs since they are equal. Specifically, *P*(*δ* = 1|**Z**) is the probability of occurrence of the event and *S_T_S_C_* = *P*(*X* > *t*|**Z**) represents the probability at risk at time t. For any t, we have

$$\frac{\partial P(\delta =1|Z)}{\partial Z_{j}}\times \frac{\partial P(X>t|Z)}{\partial Z_{j}}\leq 0.$$

By lemma 8, *Cov** {*Z_j_*, *P*(*δ* = 1|**Z**)|$Z_{C}$} and *Cov**(*Z_j_*, *S_T_S_C_*|$Z_{C}$) have the opposite signs unless they are zero. This further implies that

$$F_{2j}\left(\beta_{C,0},0\right)=\int_{0}^{\tau }\frac{E\left\{\exp\left(Z_{C}^{\text{T}}\beta_{C,0}\right)Cov^{*}\left(Z_{j},S_{T}S_{C}|Z_{C}\right)\right\}}{E\left\{\exp\left(Z_{C}^{\text{T}}\beta_{C,0}\right)S_{T}S_{C}\right\}}\times E\left\{\lambda_{0}(t)\;\exp\left(\alpha^{\text{T}}Z\right)S_{T}S_{C}\right\}\;\text{d}t,$$

and *E*[*Cov**{*Z_j_*, *P*(*δ* = 1|**Z**)|$Z_{C}$}] have opposite signs unless they are equal to zero. So $F_{2j}(\beta_{C,0},0)\neq F_{2j}\left(\beta_{C,j}^{0},\beta_{j}^{0}\right)$, therefore, $\beta_{j}^{0}\neq 0$.

Proof of Lemma 2

*Proof* By lemma 1, for any $j\in A_{C}$, we have $\beta_{j}^{0}\neq 0$. By Taylor expansion, there exists ${\tilde{\beta }}_{j}\in \left(0,\beta_{j}^{0}\right)$ such that

$$\left|v_{j}\left(\beta_{C,j}^{0},0\right)\right|=\left|v_{j}\left(\beta_{C,j}^{0},\beta_{j}^{0}\right)-v_{j}\left(\beta_{C,j}^{0},0\right)\right|=\left|\frac{\partial v_{j}}{\partial \beta_{j}}\left(\beta_{C,j}^{0},{\tilde{\beta }}_{j}\right)||\beta_{j}^{0}\right|.$$

By the proof of lemma 1, $v_{j}\left(\beta_{C,j}^{0},\beta_{j}^{0}\right)=E\left[Cov^{*}\left\{Z_{j},P(\delta =1|Z)|Z_{C}\right\}\right]-F_{2j}\left(\beta_{C,j}^{0},\beta_{j}^{0}\right)$. Given $\beta_{C,j}^{0}$, consider $F_{2j}\left(\beta_{C,j}^{0},\beta_{j}\right)$ as a function of *β_j_*, then

$$\frac{\partial F_{2j}\left(\beta_{C,j}^{0},\beta_{j}\right)}{\partial \beta_{j}}=\int_{0}^{\tau }H_{j}\left(\beta_{C,j}^{0},\beta_{j},t\right)E\left\{\lambda_{0}(t)\;\exp\left(\alpha^{\text{T}}Z\right)S_{T}S_{C}\right\}\;\text{d}t\;=E\left\{\int_{0}^{\tau }H_{j}\left(\beta_{C,j}^{0},\beta_{j},t\right)S_{C}\text{d}F_{T}(t|Z)\right\},$$

where

$$H_{j}\left(\beta_{C,j}^{0},\beta_{j},t\right)\;=\frac{E\left[Z_{j}\left\{Z_{j}-E^{*}\left(Z_{j}|Z_{C}\right)\right\}\;\exp\left(Z_{C}^{\text{T}}\beta_{C,j}^{0}+Z_{j}\beta_{j}\right)S_{T}S_{C}\right]}{E\left\{\exp\left(Z_{C}^{\text{T}}\beta_{C,j}^{0}+Z_{j}\beta_{j}\right)S_{T}S_{C}\right\}}-\frac{E\left[\left\{Z_{j}-E^{*}\left(Z_{j}|Z_{C}\right)\right\}\;\exp\left(Z_{C}^{\text{T}}\beta_{C,j}^{0}+Z_{j}\beta_{j}\right)S_{T}S_{C}\right]E\left\{Z_{j}\;\exp\left(Z_{C}^{\text{T}}\beta_{C,j}^{0}+Z_{j}\beta_{j}\right)S_{T}S_{C}\right\}}{{\left[E\left\{\exp\left(Z_{C}^{\text{T}}\beta_{C,j}^{0}+Z_{j}\beta_{j}\right)S_{T}S_{C}\right\}\right]}^{2}}$$

By condition C3, |*Z_j_*| ≤ *L*_0_, then $\underset{\beta_{j}}{\sup}\left|H_{j}\left(\beta_{C,j}^{0},\beta_{j},t\right)\right|\leq 2L_{0}^{2}$. So

$$\left|\frac{\partial v_{j}}{\partial \beta_{j}}\left(\beta_{C,j}^{0},{\tilde{\beta }}_{j}\right)\right|\leq \underset{\beta_{j}}{\sup}\left|\frac{\partial F_{2j}\left(\beta_{C,j}^{0},\beta_{j}\right)}{\partial \beta_{j}}\right|\leq 2L_{0}^{2}\left|E\left[E\left\{S_{C}(T)|Z\right\}\right]\right|\leq 2L_{0}^{2}.$$

By the proof in lemma 1, $F_{2j}\left(\beta_{C,j}^{0},0\right)\;\text{and}\;E\left[Cov^{*}\left\{Z_{j},P(\delta =1|Z)|Z_{C}\right\}\right]$ have opposite signs, combining it with condition C6,

$$\left|v_{j}\left(\beta_{C,j}^{0},0\right)\right|=\left|E\left[Cov^{*}\left\{Z_{j},P(\delta =1|Z)|Z_{C}\right\}\right]\right|+\left|F_{2j}\left(\beta_{C,j}^{0},0\right)\right|\geq c_{1}n^{-\kappa }.$$

So

$$\left|\beta_{j}^{0}\right|={\left|\frac{\partial v_{j}}{\partial \beta_{j}}\left(\beta_{C,j}^{0},{\tilde{\beta }}_{j}\right)\right|}^{-1}\left|v_{j}\left(\beta_{C,j}^{0},0\right)\right|\geq {\left(2L_{0}^{2}\right)}^{-1}c_{1}n^{-\kappa }.$$

Taking $c_{2}=0.5L_{0}^{-2}c_{1}$, we have

$$\underset{j\in A_{-C}}{\min}\left|\beta_{j}^{0}\right|\geq c_{2}n^{-\kappa },$$

which completes the proof.

Proof of Lemma 3

*Proof* Denote ${\bar{U}}_{j}\left(\beta_{C,j},\beta_{j}\right)=n^{-1}U_{j}\left(\beta_{C,j},\beta_{j}\right)$. By the definition of ${\left({\hat{\beta }}_{C,j},{\hat{\beta }}_{j}\right)}^{\text{T}}$, we have

$$\left\|{\bar{U}}_{j}\left({\hat{\beta }}_{C,j},{\hat{\beta }}_{j}\right)-{\bar{U}}_{j}\left(\beta_{C,j}^{0},\beta_{j}^{0}\right)\right\|=\left\|{\bar{U}}_{j}\left(\beta_{C,j}^{0},\beta_{j}^{0}\right)\right\|.$$

For any j∉C and k∈C∪{j}, using the similar method of Lin and Wei (1989), by lemmas 4 and 5, we can obtain that

$${\bar{U}}_{j}\left(\beta_{C,j}^{0},\beta_{j}^{0}\right)=n^{-1}\overset{n}{\underset{i=1}{\sum }}W_{i,j}\left(\beta_{C,j}^{0},\beta_{j}^{0}\right)+o_{p}(1),$$

where $W_{i,j}\left(\beta_{C,j}^{0},\beta_{j}^{0}\right)\;(i=1,\ldots ,n)$ are independent, $E\left\{W_{i,j}\left(\beta_{C,j}^{0},\beta_{j}^{0}\right)\right\}=0\;\text{and}\;W_{i,j}\left(\beta_{C,j}^{0},\beta_{j}^{0}\right)=[W_{i,j,k}\left(\beta_{C,j}^{0},\beta_{j}^{0}\right)$, and $W_{i,j}\left(\beta_{C,j}^{0},\beta_{j}^{0}\right)=[W_{i,j,k}\left(\beta_{C,j}^{0},\beta_{j}^{0}\right),k\in C\cup \{j\}{]}^{\text{T}}$ with

$$W_{i,j,k}\left(\beta_{C,j}^{0},\beta_{j}^{0}\right)\;=\int_{0}^{\tau }\left[Z_{ik}-\frac{E\left\{Z_{ik}\;\exp\left(\beta_{C,j}^{0}Z_{i,C}+\beta_{j}^{0}Z_{ij}\right)S_{T}S_{C}\right\}}{E\left\{\exp\left(\beta_{C,j}^{0}Z_{i,C}+\beta_{j}^{0}Z_{ij}\right)S_{T}S_{C}\right\}}\right]\;\text{d}N_{i}(t)\;-\int_{0}^{\tau }\frac{Y_{i}(t)\;\exp\left(\beta_{C,j}^{0}Z_{i,C}+\beta_{j}^{0}Z_{ij}\right)}{E\left\{\exp\left(\beta_{C,j}^{0}Z_{i,C}+\beta_{j}^{0}Z_{ij}\right)S_{T}S_{C}\right\}}\left[Z_{ik}-\frac{E\left\{Z_{ik}\;\exp\left(\beta_{C,j}^{0}Z_{i,C}+\beta_{j}^{0}Z_{ij}\right)S_{T}S_{C}\right\}}{E\left\{\exp\left(\beta_{C,j}^{0}Z_{i,C}+\beta_{j}^{0}Z_{ij}\right)S_{T}S_{C}\right\}}\right]\;E\left\{\text{d}N_{i}(t)\right\}.$$

Let *E_n_* denote the empirical measure, we can write

$${\bar{U}}_{j}\left(\beta_{C,j}^{0},\beta_{j}^{0}\right)=E_{n}\left[W_{i,j}\left(\beta_{C,j}^{0},\beta_{j}^{0}\right)\right]+o_{p}(1).$$

For any given *i, j, k,* by conditions C1, C3, C5, there exists a constant *L_2_* such that $\left|W_{i,j,k}\left(\beta_{C,j}^{0},\beta_{j}^{0}\right)\right|\leq L_{2}$, by the fact that $E\left[W_{i,j,k}\left(\beta_{C,j}^{0},\beta_{j}^{0}\right)\right]=0$, we have $Var\left[W_{i,j,k}\left(\beta_{C,j}^{0},\beta_{j}^{0}\right)\right]=E\left[{\left|W_{i,j,k}\left(\beta_{C,j}^{0},\beta_{j}^{0}\right)\right|}^{2}\right]\leq L_{2}^{2}$. By lemma 6, for any *t* > 0, j∉C and k∈C∪{j}, we have

$$P\left(\left|E_{n}\left(W_{i,j,k}\left(\beta_{C,j}^{0},\beta_{j}^{0}\right)\right)\right|>\frac{t}{n}\right)\leq 2\;\exp\;\left(-\frac{1}{2}\frac{t^{2}}{nL_{2}^{2}+L_{2}t/3}\right).$$

By Bonferroni inequality, we have

$$P\left({\left\|E_{n}\left(W_{i,j}\left(\beta_{C,j}^{0},\beta_{j}^{0}\right)\right)\right\|}_{2}>\frac{t(q+1)}{n}\right)\leq 2(q+1)\;\exp\;\left(-\frac{1}{2}\frac{t^{2}}{nL_{2}^{2}+L_{2}t/3}\right).$$

As ${\left\|{\bar{U}}_{j}\left(\beta_{C,j}^{0},\beta_{j}^{0}\right)-E_{n}\left[W_{i,j}\left(\beta_{C,j}^{0},\beta_{j}^{0}\right)\right]\right\|}_{2}=o_{p}(1),$, for any *ϵ*_1_ > 0 and *ϵ*_2_ > 0, there exists *N*_1_ such that for

$$P\left({\left\|{\bar{U}}_{j}\left(\beta_{C,j}^{0},\beta_{j}^{0}\right)-E_{n}\left[W_{i,j}\left(\beta_{C,j}^{0},\beta_{j}^{0}\right)\right]\right\|}_{2}>Lc_{2}\epsilon_{1}/2\right)<\epsilon_{2}.$$

Taking $t=\frac{c_{2}\;Ln^{1-\kappa }}{2(q+1)}>0$, then $\frac{t(q+1)}{n}=\frac{c_{2}Ln^{-\kappa }}{2}$. By Triangle inequality and Bonferroni inequality, we have

$$P\left({\left\|{\bar{U}}_{j}\left(\beta_{C,j}^{0},\beta_{j}^{0}\right)\right\|}_{2}>Lc_{2}\left(n^{-\kappa }+\epsilon_{1}\right)/2\right)\;\leq P\left({\left\|E_{n}\left\{W_{i,j}\left(\beta_{C,j}^{0},\beta_{j}^{0}\right)\right\}\right\|}_{2}>Lc_{2}n^{-\kappa }/2\right)\;+P\left({\left\|{\bar{U}}_{j}\left(\beta_{C,j}^{0},\beta_{j}^{0}\right)-E_{n}\left\{W_{i,j}\left(\beta_{C,j}^{0},\beta_{j}^{0}\right)\right\}\right\|}_{2}>Lc_{2}\epsilon_{1}/2\right)\;\leq 2(q+1)\;\exp\left(-\frac{1}{2}\frac{c_{2}^{2}L^{2}n^{2-2\kappa }/4{\left(q+1\right)}^{2}}{nL_{2}^{2}+L_{2}c_{2}Ln^{1-\kappa }/6(q+1)}\right)+\epsilon_{2}.$$

Taking *N* = max{(*L*_2_/3)^1/*κ*^, *N*^1^}, then for any *n* > *N*, *n^−κ^* < 3/*L*_2_, so we have

$$P\left({\left\|{\bar{U}}_{j}\left(\beta_{C,j}^{0},\beta_{j}^{0}\right)\right\|}_{2}>Lc_{2}\left(n^{-\kappa }+\epsilon_{1}\right)/2\right)\leq 2(q+1)\;\exp\;\left(-c_{3}n^{1-2\kappa }\right)+\epsilon_{2},$$

where $c_{3}=\frac{c_{2}^{2}L^{2}}{8L_{2}^{2}{\left(q+1\right)}^{2}+4c_{2}L(q+1)}$. By condition C7, we have

$$P\left(\left|{\hat{\beta }}_{j}-\beta_{j}^{0}\right|>c_{2}\left(n^{-\kappa }+\epsilon_{1}\right)/2\right)\;\leq P\left({\left\|{\left({\hat{\beta }}_{C,j},{\hat{\beta }}_{j}\right)}^{\text{T}}-{\left(\beta_{C,j}^{0},\beta_{j}^{0}\right)}^{\text{T}}\right\|}_{2}>c_{2}\left(n^{-\kappa }+\epsilon_{1}\right)/2\right)\;\leq P\left({\left\|{\bar{U}}_{j}{\left({\hat{\beta }}_{C,j},{\hat{\beta }}_{j}\right)}^{\text{T}}-{\bar{U}}_{j}\left(\beta_{C,j}^{0},\beta_{j}^{0}\right)\right\|}_{2}>Lc_{2}\left(n^{-\kappa }+\epsilon_{1}\right)/2\right)\;=P\left({\left\|{\bar{U}}_{j}\left(\beta_{C,j}^{0},\beta_{j}^{0}\right)\right\|}_{2}>Lc_{2}\left(n^{-\kappa }+\epsilon_{1}\right)/2\right)\;\leq 2(q+1)\;\exp\;\left(-c_{3}n^{1-2\kappa }\right)+\epsilon_{2}.$$

Then we have

$$P\left(\underset{j\in A_{-C}}{\max}\left|{\hat{\beta }}_{j}-\beta_{j}^{0}\right|>c_{2}\left(n^{-\kappa }+\epsilon_{1}\right)/2\right)\leq 2a(q+1)\;\exp\left(-c_{3}n^{1-2\kappa }\right)+a\epsilon_{2},$$

where $a=\left|A_{-C}\right|=\sum_{j\notin C}\;I\left(\alpha_{j}\neq 0\right)$ is the size of |$A_{-C}$|.

Proof of Theorem 1

*Proof* By the definition of $A_{-C}$ and condition C7, there exists a positive constant *c*_4_ such that

$$P\left(A_{-C}\subseteq {\hat{A}}_{-C}\right)=P\left(\underset{j\in A_{-C}}{\min}\left|{\hat{\beta }}_{j}\right|/{\hat{\sigma }}_{j}\geq \gamma \right)\geq 1-P(\underset{j\in A_{-C}}{\min}\left|{\hat{\beta }}_{j}\right|<n^{-1/2}c_{4}\gamma ).$$

Following lemma 2, for any $j\in A_{-C}$, we have $\left|\beta_{j}^{0}-{\hat{\beta }}_{j}\right|\geq \left|\beta_{j}^{0}\right|-\left|{\hat{\beta }}_{j}\right|\geq c_{2}n^{-\kappa }-\left|{\hat{\beta }}_{j}\right|$. Suppose $\min_{j\in A_{-C}}|{\hat{\beta }}_{j}|$ < *n*^−1/2^*c*_4_*γ*, then $\underset{j\in A_{-C}}{\max}\left|\beta_{j}^{0}-{\hat{\beta }}_{j}\right|\geq c_{2}n^{-\kappa }-n^{-1/2}c_{4}\gamma$. If we have $\gamma <c_{2}\left(n^{-\kappa }-\epsilon_{1}\right)n^{1/2}/\left(2c_{4}\right)$, we can obtain

$$P\left(\underset{j\in A_{-C}}{\min}\left|{\hat{\beta }}_{j}\right|<n^{-1/2}c_{4}\gamma \right)<P(\underset{j\in A_{-C}}{\max}\left|\beta_{j}^{0}-{\hat{\beta }}_{j}\right|\geq c_{2}\left(n^{-\kappa }+\epsilon_{1}\right)/2).$$

Then $P\left(A_{-C}\subseteq {\hat{A}}_{-C}\right)\geq 1-2a(q+1)\;\exp\left(-c_{3}n^{1-2\kappa }\right)-a\epsilon_{2}$. Let *n* → ∞, for any *ϵ*_2_ > 0, we have $\lim_{n\to \infty }P(A_{-C}\subseteq {\hat{A}}_{-C})\geq 1-a\epsilon_{2}$, the right side of the above equation does not depend on *n* any more. Taking *ϵ*_2_ → 0, we have $\lim_{n\to \infty }P(A_{-C}\subseteq {\hat{A}}_{-C})=1$.

Proof of Theorem 2

*Proof* For any $j\in A_{-C}$, we have *α_j_* ≠ 0. From lemma 1, we know that $\left|\beta_{j}^{0}\right|>0$. Similarity, we have $|\beta_{j}^{0}|>0$. Similarly, we have $|\beta_{j}^{0}|=0$ if $j\in A_{-C}$. As ${\hat{\beta }}_{j}$ is a consistent estimator of $\beta_{j}^{0}$ and $M_{C,j}=\left|{\hat{\beta }}_{j}\right|/{\hat{\sigma }}_{j}$, we can easily conclude that $P(\max_{j\notin A_{-C}}\;M_{C,j}<\min_{j\in A_{-C}}\;M_{C,j})\to 1$ when *n* → ∞, which completes the proof of theorem 2.
