# Supplementary material for: Conditional screening for ultrahigh-dimensional survival data in case-cohort studies
Source: Lifetime Data Anal. Author manuscript; Available in PMC 2022 Oct 1. (PMC8561435; doi:10.1007/s10985-021-09531-7)
Supplement: 1745068_Sup_material [file NIHMS1745068-supplement-1745068_Sup_material.pdf]

Noname manuscript No.
(will be inserted by the editor)

Supplementary material for "Conditional screening for ultrahigh-dimensional survival data in case-cohort studies"

Received: date / Accepted: date

This supplementary material contains some additional results for real data analysis.

We further consider $d_n = n/2, n/3, n/4$ in real data analysis and denote them as cases (a)–(c). Specially, we first reduce the dimension from p to d_n and then followed with regularization methods LASSO, SCAD and MCP. The names of selected genes and their corresponding parameter estimates are summarized in Tables S1–S3 below, respectively, from which we can see that the selected genes under different cut-offs are highly consistent. In particular, six genes Contig58368.RC, NM.003920, NM.014889, Contig63649.RC, Contig11075.RC, AL080059 were all selected by the LASSO, SCAD and MCP methods for case (a) with $d_n = n/2$; eight genes Contig58368.RC, NM.003920, NM.014889, Contig63649.RC, AL080059, NM.013332,NM.003752, NM.013290 were all selected for case (b) with $d_n = n/3$; the same eight genes were all selected for case (c) with $d_n = n/4$. We further compute the C-statistic estimator for CWSIS, MWSIS, NCWSIS procedures for $d_n = n/2, n/3, n/4$, the results are summarized in Table S4 below, from which we can make similar conclusion to that with $d_n = n/\log(n)$.

Table S1 The results of selected important genes for the breast cancer data using the regularization methods for case (a) ($d_n = n/2$)

LASSO		SCAD		MCP	
Name	Est.	Name	Est.	Name	Est.
Contig58368.RC	0.3885	NM.003920	0.6020	Contig58368.RC	0.5939
NM.014889	0.2396	Contig58368.RC	0.5966	NM.003920	0.5008
Contig63649.RC	0.2341	NM.014889	0.4720	NM.014889	0.4923
NM.003920	0.2159	Contig63649.RC	0.4417	Contig63649.RC	0.3616
Contig11075.RC	0.1608	Contig11075.RC	0.2756	Contig11075.RC	0.1635
NM.013332	0.1356	Contig46991.RC	-0.1454	AL080059	0.1099
AL080059	0.1348	AL080059	0.1231		
Contig46991.RC	-0.1247	Contig32050.RC	0.0429		
NM.005689	0.1098	NM.013332	0.0181		
Contig31288.RC	0.0977				
NM.013290	0.0760				
NM.002808	0.0698				
Contig32050.RC	0.0572				
NM.003430	-0.0538				
NM.014214	0.0474				
NM.003752	0.0425				
Contig45729.RC	0.0285				
NM.006579	0.0139				
NM.003376	0.0135				
NM.006461	0.0020				
AB014568	0.0002				

Name: the name for selected genes; Est.: the corresponding estimated value of the coefficient for selected genes.

Table S2 The results of selected important genes for the breast cancer data using the regularization methods for case (b) ($d_n = n/3$)

LASSO		SCAD		MCP	
Name	Est.	Name	Est.	Name	Est.
Contig58368.RC	0.3985	Contig58368.RC	0.6112	Contig58368.RC	0.6307
NM.014889	0.2883	NM.014889	0.5166	NM.003920	0.5582
Contig63649.RC	0.2041	NM.003920	0.4932	NM.014889	0.5164
AL080059	0.1919	Contig63649.RC	0.4040	Contig63649.RC	0.4059
NM.003920	0.1792	AL080059	0.2472	AL080059	0.2793
NM.013332	0.1689	NM.013332	0.0714	NM.013332	0.1091
NM.005689	0.1661	NM.013290	0.0676	NM.003752	0.0746
NM.013290	0.1639	NM.003752	0.0672	NM.013290	0.0089
NM.003752	0.1090	NM.001333	0.0264		
NM.002916	0.0862	NM.005689	0.0262		
NM.006579	0.0611	AL160131	0.0127		
NM.001605	0.0427				
NM.003376	0.0273				
Contig31288.RC	0.0255				
NM.001333	0.0216				
NM.014214	0.0206				
Contig38288.RC	0.0149				

Name: the name for selected genes; Est.: the corresponding estimated value of the coefficient for selected genes.

Table S3 The results of selected important genes for the breast cancer data using the regularization methods for case (c) ($d_n = n/4$)

LASSO		SCAD		MCP	
Name	Est.	Name	Est.	Name	Est.
Contig58368.RC	0.4241	Contig58368.RC	0.6268	Contig58368.RC	0.6255
NM.014889	0.3237	NM.014889	0.5380	NM.003920	0.5347
NM.003920	0.2115	NM.003920	0.5316	NM.014889	0.5326
AL080059	0.2059	Contig63649.RC	0.3980	Contig63649.RC	0.3806
Contig63649.RC	0.1989	AL080059	0.2498	AL080059	0.2973
NM.013332	0.1895	NM.013332	0.0841	NM.013332	0.1469
NM.005689	0.1795	NM.013290	0.0656	NM.013290	0.0497
NM.013290	0.1691	NM.005689	0.0212	NM.005689	0.0309
NM.002916	0.0977				
Contig31288.RC	0.0571				
NM.014214	0.0545				
Contig38288.RC	0.0346				
NM.003376	0.0338				
NM.001673	0.0016				
NM.000270	0.0001				

Name: the name for selected genes; Est.: the corresponding estimated value of the coefficient for selected genes.

Table S4 The C-statistic and standard deviation (SD) of the five screening methods for the diffuse large-B-cell lymphoma (DLBCL) dataset

Case		CWSIS	MWSIS	NCWSIS
(a)	C-statistic	0.917	0.811	0.903
	Standard deviation	0.130	0.078	0.087
(b)	C-statistic	0.886	0.780	0.881
	Standard deviation	0.081	0.090	0.061
(c)	C-statistic	0.867	0.786	0.851
	Standard deviation	0.062	0.083	0.063

CWSIS: the proposed screening procedure; MWSIS: the marginal weighted screening procedure; NCWSIS: the unweighted conditional screening method; case (a): $d_0 = n/2$; case (b): $d_0 = n/3$; case (c): $d_0 = n/4$.
